# The ERF Transcription Factor *ERF41* Negatively Regulates Drought and Salt Tolerance in *Arabidopsis thaliana*

**DOI:** 10.3390/life16030421

**Published:** 2026-03-04

**Authors:** Jing Wang, Mengli Luo, Han Xiao, Yue Zhang

**Affiliations:** Shandong Key Laboratory for Germplasm Innovation of Saline-Alkaline Tolerant Grasses and Trees, College of Grassland Science, Qingdao Agricultural University, Qingdao 266109, China; 15104937626@163.com (J.W.); 20232252014@stu.qau.edu.cn (M.L.); 18266391263@163.com (H.X.)

**Keywords:** *Arabidopsis thaliana*, salt tolerance, drought tolerance, transcription factor, DREB

## Abstract

Drought and salt stresses severely impair plant growth and development worldwide. DEHYDRATION-RESPONSIVE ELEMENT BINDING proteins (DREBs), as a subfamily of the AP2/ERF transcription factor superfamily, play critical regulatory roles in plant biological processes including growth and development, as well as the adaptive response to various abiotic stresses. Based on the transcriptome data analysis of *Medicago truncatula* under saline-alkali stress previously conducted in our laboratory, a gene responsive to saline-alkali stress, Medtr3g110205, was identified, and its homologous gene in *Arabidopsis thaliana*, *AtERF41* (AT5G11590), was obtained via BLAST (version BLAST+ 2.17.0.). The mutant *erf41* was used to explore its biological functions in response to drought and salt stresses. The results showed that under salt and drought stress conditions, the seed germination rate, and growth status of the *erf41* mutant were all better than those of the wild type. Further determination of physiological and biochemical indicators revealed that the leaf contents of superoxide dismutase (SOD) and proline (Pro) in the leaves of the mutant plants were significantly higher than those in the wild type, while the malondialdehyde (MDA) content was significantly decreased. In conclusion, the *AtERF41* gene negatively regulates salt and drought tolerance in *Arabidopsis thaliana*, providing a potential target for the genetic improvement of crop stress tolerance. This study not only deepens our understanding of the role of DREB transcription factors in plant stress response but also provides a theoretical basis for improving crop stress tolerance using genetic engineering technology in the future.

## 1. Introduction

Dynamic environmental conditions such as drought and high salinity exert adverse effects on plant growth, survival, and reproduction [1]. Drought and high salt concentrations induce osmotic stress in plant cells and cause the accumulation of harmful ions, thereby leading to plant stunting or developmental retardation [2]. Additionally, drought and salt stress can induce the accumulation of reactive oxygen species (ROS), such as superoxide anions, hydrogen peroxide, and hydroxyl radicals. ROS possess strong oxidative activity that attacks cellular membranes and intracellular membrane systems, causing plasma membrane peroxidation, and leading to cell membrane damage and cell death, thereby impairing plant growth and development [3]. During long-term evolutionary adaptation, plants have developed corresponding mechanisms to cope with drought and salt stress, mitigating cellular damage to maintain survival under stressful conditions [4]. Notably, the AP2/ERF transcription factors are indispensable in mediating plant responses to osmotic stress [4].

AP2/ERF transcription factors are key regulators in plants, participating in the modulation of plant growth and development, biosynthesis, and stress responses [5]. AP2/ERF contains 1–2 AP2/ERF domains, which consist of approximately 60–70 conserved amino acids involved in DNA binding. Based on the number of AP2/ERF domains and their binding sequences, AP2/ERF transcription factors are classified into five subfamilies: AP2, ERF, DREB, RAV, and Soloist [6,7]. Specifically, the AP2 subfamily comprises two AP2/ERF domains, whereas the ERF and DREB subfamilies each harbor a single AP2/ERF domain. The RAV subfamily contains one AP2/ERF domain together with one B3 domain; while the Soloist subfamily also contains one AP2/ERF domain but exhibits significant structural differences from other subfamilies [8]. Studies have demonstrated that overexpression of *OsERF19* enhances salt tolerance in rice [9]. *GmERF75* is induced by multiple abiotic stresses and exogenous phytohormones, and overexpression of this gene improves osmotic stress tolerance in both *Arabidopsis* and soybean [10]. Similarly, in rice, *OsERF115/AP2EREBP110* has been found to be induced by high-temperature and drought treatments; its overexpression enhances the heat tolerance of plants at both the seed and vegetative growth stages, and improves plant drought tolerance by increasing the accumulation of osmotic regulators [11]. Additionally, research indicates that overexpression of GmDREB1 markedly enhances drought tolerance in soybean [12].

Previous studies have identified 147 AP2/ERF genes in *Arabidopsis thaliana*, of which 122 belong to the ERF (including DREB) subfamily; among these 122 ERF genes, 57 are members of the DREB subfamily [13]. TINY is a member of the DEHYDRATION-RESPONSIVE ELEMENT BINDING protein A4 subfamily of the AP2/ERF transcription factor family, which comprises 17 members in *Arabidopsis*, and both *TINY* and *ERF41* (also known as *TINY2*) are classified into this clade [13]. Studies have demonstrated that *TINY* transcript levels are strongly induced by multiple stresses, including dehydration, cold, and salt. Additionally, TINY overexpression is associated with elevated expression of drought-responsive genes, as well as hypersensitivity to ABA-mediated inhibition of seed germination and root growth [14,15,16]. Wei et al. demonstrated that *TINY2* is induced by ABA, cold, wounding, NaCl, and drought treatments, suggesting that *TINY2* may be involved in activating downstream genes in response to environmental stress [17].

In conclusion, we speculate that *ERF41* plays a role in plant responses to drought and salt stresses, but its specific function remains unclear. To explore the function of *AtERF41* in plant responses to drought and salt stress, we utilized the *Arabidopsis* mutant *erf41* as experimental material to study the function of the *ERF41* gene, aiming to provide theoretical insights for understanding the mechanisms of plant responses to drought and salt stress.

## 2. Materials and Methods

### 2.1. Plant Materials and Growth Conditions

The wild-type *Arabidopsis thaliana* was purchased from ABRC (*Arabidopsis* Biological Resource Center) as the Columbia-0 (*Col-0*) line, and the mutant *erf41* (N690869) was obtained from the Arashare website. *Arabidopsis* seeds were first rinsed twice with water, then treated with 75% alcohol for 1 min, followed by exposure to 1% NaClO for 10 min, and finally washed five times with distilled water. Seeds were sown on 1/2MS medium plates containing 30 g/L sucrose and 6 g/L agar. The seeded plates were vernalized at 4 °C in the dark for 2 days and then moved to 22 °C under a 16/8 light/dark cycle. When *Arabidopsis* sprouted, it was transplanted at a density of four plants per pot with a mixture of soil at 22 °C under a 16/8 light/dark cycle and 70% relative humidity.

### 2.2. Analysis of the Physicochemical Properties and Conserved Domains of AtERF41

The physicochemical properties of the AtERF41 protein were analyzed using the ExPASY ProtParam tool (https://web.expasy.org/protparam/, accessed on 3 February 2026). The transmembrane regions, hydrophilicity, and signal peptides of the protein were predicted using TMHMM 2.0 Server (https://services.healthtech.dtu.dk/services/TMHMM-2.0/, accessed on 3 February 2026), ExPASY Protscale (https://web.expasy.org/protscale/, accessed on 3 February 2026), and SignalP 4.1 Server (https://services.healthtech.dtu.dk/services/SignalP-4.1/, accessed on 3 February 2026), respectively. The secondary and tertiary structures of the protein were predicted using the online SOPMA (https://npsa-prabi.ibcp.fr/cgi-bin/npsa_automat.pl?page=npsa_sopma.html, accessed on 3 February 2026) and SWISS-MODEL (https://swissmodel.expasy.org/, accessed on 3 February 2026) tools, respectively. The conserved domains of the amino acid sequences encoded by the gene were analyzed using the Conserved Domain Database (https://www.ncbi.nlm.nih.gov/cdd/?term=, accessed on 3 February 2026).

### 2.3. Identification of Arabidopsis erf41 Mutant Homozygotes

The *Arabidopsis thaliana* mutant seeds were cultured on 1/2 MS medium as previously described. After seedling emergence, homozygosity was confirmed by PCR.

Homozygous mutation identification was performed using the three-primer method. The LP, RP, and BP primers for the mutants were obtained from the website (http://signal.salk.edu/tdnaprimers.2.html?tdsourcetag=s_pcqq_aiomsg, accessed on 3 February 2026) for PCR detection (Table 1). PCR was conducted using the ExTaq enzyme (Aidlab Biotech, Beijing, China) with the primers LP + RP and BP + RP. The principle is as follows: the primers LP + RP and BP + RP can simultaneously amplify products of a larger molecular weight product (large fragments of the wild-type gene) and a smaller molecular weight product (small fragments of T-DNA insertion). In homozygous mutants, only the BP + RP primer can amplify the smaller molecular weight product, while the LP + RP primer fails to produce any product.

### 2.4. Quantitative Real-Time PCR Analysis

RNA was extracted from the leaves of *Col-0* and *erf41* plants using the Plant Total RNA purification kit with DNase I (Aidlab), followed by the synthesis of complementary DNA (cDNA) using the Tiangen FastQuant RT Kit (Tiangen, Beijing, China) following the manufacturer’s instructions. Subsequently, quantitative real-time PCR (RT-qPCR) was performed using SYBR Green fluorescence detection. For normalization, *Arabidopsis Actin* was used as an internal standard. Five biological replicates × four technical replicates were used for qRT-PCR to ensure the reproducibility of the results. The primers are listed in Table 2.

### 2.5. Physiological Experiments

To determine germination rates of *Col-0* and mutant *erf41* seeds, 90 (30 biological replicates × 3 technical replicates) seeds from each genotype (*Col-0* and mutant *erf41*) were sown individually on the same 1/2MS medium supplemented with or without NaCl (100 mM, 125 mM, 150 mM) and mannitol (200 mM, 300 mM), with daily recordings of germination rates. Following germination, 15 (5 biological replicates × 3 technical replicates) seedlings per genotype were selected and vertically cultured on medium with or without NaCl stress (100 mM, 125 mM, 150 mM) for 10 days, after which root lengths were measured.

To measure the activity of ROS-scavenging enzymes, *Arabidopsis thaliana* leaves were collected before and after treatment. Then, soluble proteins were extracted using physiological salt buffer, and the activities of MDA, SOD, Pro, and catalase (CAT) were measured using a kit from the Nanjing Jiancheng Bioengineering Institute (Nanjing, China). Here, 45 replicates (15 biological replicates × 3 technical replicates) were performed in each experiment.

### 2.6. Salt and Drought Experiments

For salt stress treatment, *Col-0* and *erf41* mutant *Arabidopsis* seeds were sown on 1/2 MS medium. After developing two true leaves, the seedlings were transplanted into pots (four plants per pot) containing a soil mixture, and cultured in a greenhouse at 22 °C with a 16/8 h light/dark cycle and 70% relative humidity. The seedlings were watered normally for three weeks, after which they were irrigated with a 300 mM NaCl solution every 3 days for 15 days to observe phenotypic changes.

For the drought stress tolerance test, plants were grown in well-watered soil for three weeks before water was withheld until phenotypic differences appeared among the genotypes.

### 2.7. Statistical Analysis of Data

Statistical analysis of experimental data was carried out using SPSS17.0 software. One-way ANOVA with Tukey–Kramer multiple comparisons tests was performed on the relative gene expression. Two-way ANOVA with Tukey–Kramer multiple comparisons was used for the analysis of seed germination rate, root length, SOD activity, MDA content, and Pro content. Image editing and processing were performed using Photoshop.

## 3. Results

### 3.1. Physicochemical Properties and Conserved Domains Analysis of AtERF41

**Figure 1:**
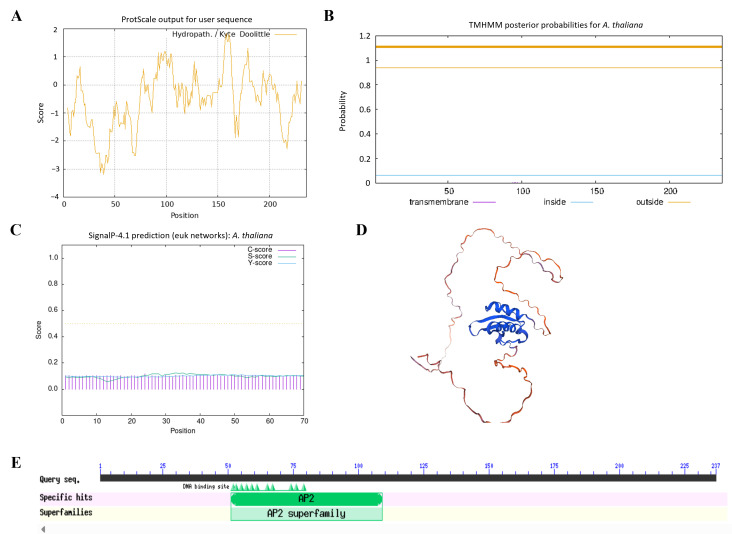
Physicochemical properties and conserved domains analysis of AtERF41. Hydrophilic and hydrophobic (**A**), transmembrane structure (**B**), signal peptide prediction (**C**), tertiary structure prediction (**D**), conserved domain analysis (**E**) of the AtERF41 protein.

To investigate the structural characteristics of *AtERF41*, the physicochemical properties of the protein encoded by the *AtERF41* gene were analyzed using ProtParam. The results indicated that this gene encodes a protein consisting of 236 amino acids, with the molecular formula C_1143_H_1777_N_315_O_370_S_6_, a molecular weight of 26.043 kDa, and an isoelectric point (pI) of 5.32. Its instability index was calculated as 54.34, classifying it as an unstable protein (proteins with an instability index below 40 are considered stable). Hydrophilicity-hydrophobicity analysis revealed a grand average of hydropathicity (GRAVY value of −0.531), indicating that this protein is hydrophilic (Figure 1A). Transmembrane structure prediction using TMHMM showed that the protein encoded by *ERF41* lacks transmembrane helical regions (Figure 1B). Analysis with the SignalP 4.1 Server demonstrated that this protein does not contain a signal peptide, suggesting that it is a non-secretory protein (Figure 1C). Secondary structure prediction using the SOPMA online tool revealed that the protein is primarily composed of α-helices (25.85%), random coils (68.22%), and extended chains (5.93%), among which random coils accounted for the highest proportion. The tertiary structure of the protein encoded by *MsERF41* predicted by SWISS-MODEL was consistent with the secondary structure prediction results (Figure 1D). Conserved domain analysis of the ERF41 protein using the Conserved Domain Database (CDD) identified one conserved AP2 domain, confirming that this gene belongs to the ERF transcription factor family (Figure 1E).

### 3.2. Identification and Expression Analysis of Arabidopsis erf41 Mutant Homozygotes

**Figure 2:**
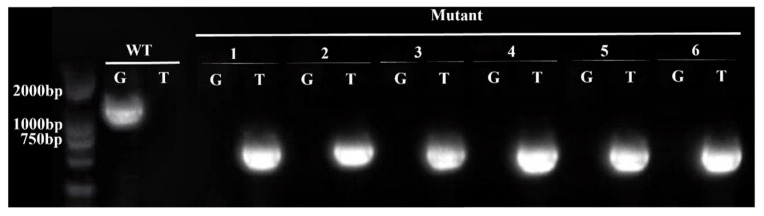
Identification of homozygous *erf41* mutant plants in *Arabidopsis thaliana*.

DNA was extracted from the *Arabidopsis thaliana* mutant *erf41* and *Col-0* plants, followed by PCR amplification using LP + RP and BP + RP as primer pairs (Table 1), with the expected band sizes of 1194 bp and 519 bp, respectively. As shown in Figure 2, agarose gel electrophoresis results confirmed that lines 1, 2, 3, 4, 5, and 6 were homozygous mutant plants based on the principle of the three-primer method. Seeds of the verified homozygous mutants were harvested, dried, and then stored in a dry place.

**Figure 3:**
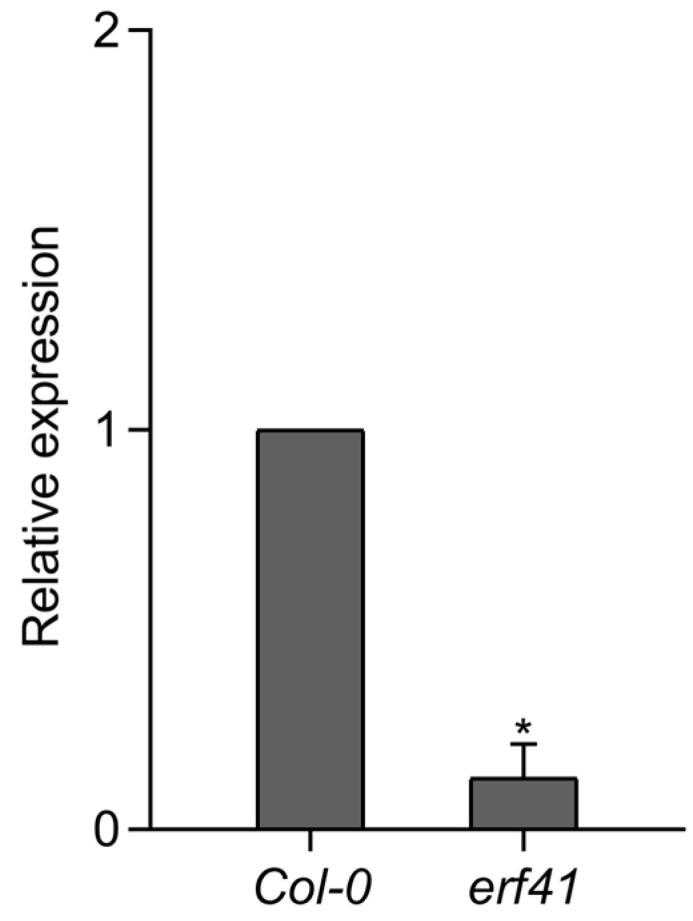
Expression analysis of the *AtERF41* in *Col-0* and *erf41* mutant plants. Data are presented as means ± SE (*n* = 5 biological replicates per genotype). The asterisks (*) represent significant differences (* *p* < 0.05).

Next, to analyze the expression of the *ERF41* gene in the *erf41* mutant plants, RT-qPCR was performed on leaves of *Col-0* and mutant plants. The results showed that *ERF41* was barely expressed in the *erf41* mutant plants, indicating that the *ERF41* gene was disrupted and thus failed to function normally (Figure 3).

### 3.3. ERF41 Negatively Regulates Salt Tolerance in Arabidopsis thaliana

**Figure 4:**
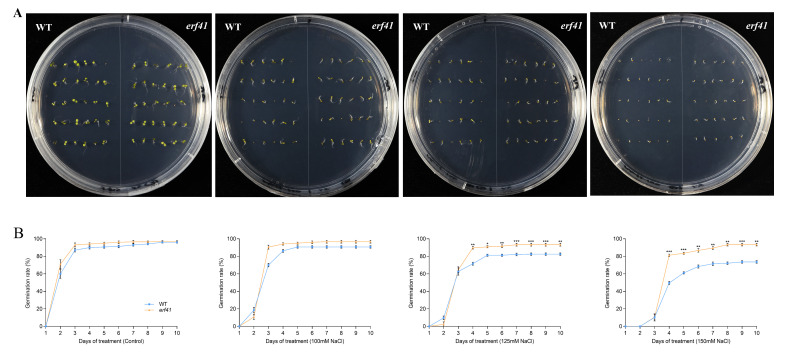
Germination rate of wild-type and *erf41* mutant plants under salt stress. Germination performance (**A**) and germination rate (**B**) of wild-type and mutant seeds under salt stress. The data are represented as means ± SE (*n* = 30 biological replicates per genotype). The asterisks (*, ** and ***) represent significant differences (* *p* < 0.05, ** *p* < 0.01, and *** *p* < 0.001) compared with WT plants at the same time point.

**Figure 5:**
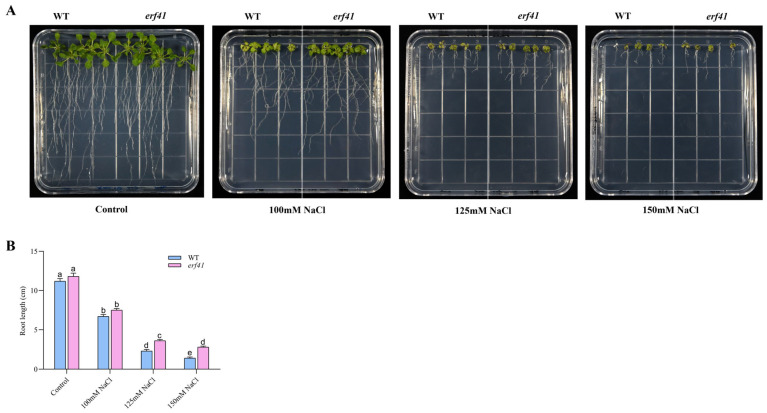
Morphological analysis of roots from wild-type and *erf41*mutant plants grown on 1/2 MS medium with or without NaCl. (**A**) Morphology analysis. (**B**) The primary root length. The data are represented as means ± SE (*n* = 5 biological replicates per genotype). Different letters represent significant differences (*p *< 0.05) compared to the control.

A series of experiments were performed to examine the differences in salt tolerance of *Col-0* and *erf41* plants. The seeds of *Col-0* and *erf41* were sown on 1/2 MS culture containing 100 mM, 125 mM, and 150 mM NaCl to observe germination rates. The results showed that on normal 1/2 MS medium, there was no significant difference in the seed germination rate among different *Arabidopsis thaliana* genotypes, all of which were close to 100%. Under the treatments of 100 mM, 125 mM and 150 mM NaCl, the germination rates of *Col-0* seeds were 90.7%, 82.5% and 73.6%, respectively, while those of *erf41* seeds were 96.7%, 93.3% and 93.3%, which were significantly higher than those of *Col-0* seeds (Figure 4A,B). In addition, the primary root length of the seedlings differed after 10 days of vertical growth under salt stress. Results showed no significant difference in root length among genotypes on normal 1/2 MS medium. However, in salt-treated conditions, *erf41* plants exhibited significantly longer roots than *Col-0* plants (Figure 5A,B). It was preliminarily inferred that *ERF41* plays a role under salt stress and negatively regulates the salt tolerance of *Arabidopsis thaliana*.

**Figure 6:**
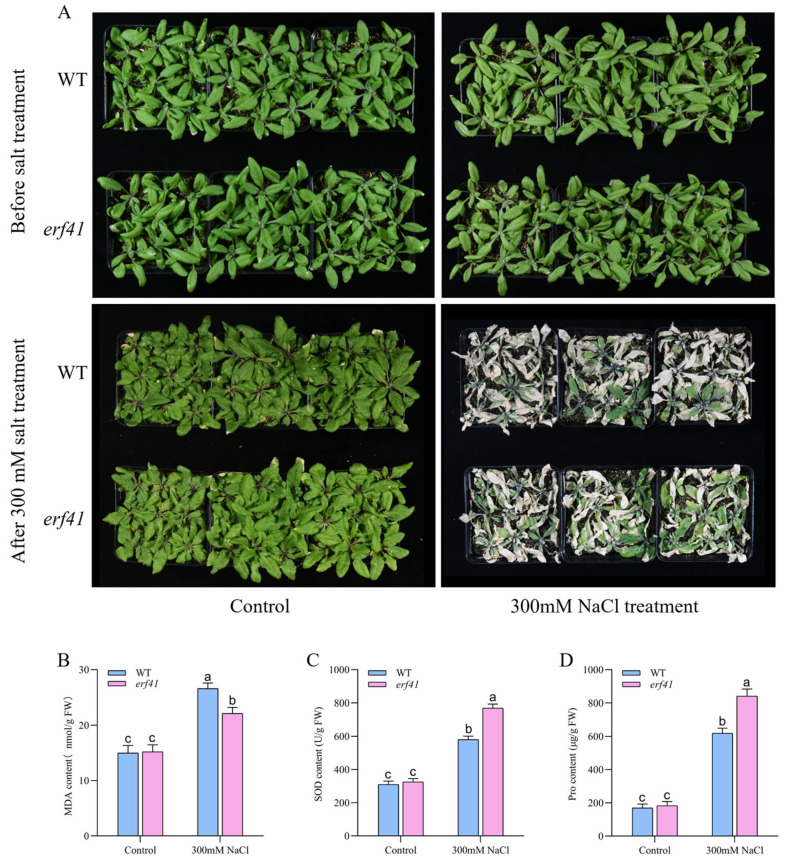
*AtERF41* negatively regulates salt tolerance in *Arabidopsis thaliana*. (**A**) Morphological differences in plants under salt experiments. The content of MDA (**B**), SOD (**C**), Pro (**D**). Data are means ± SE (*n* = 15 biological replicates per genotype). Different letters denote significant differences: *p* < 0.05.

To further investigate the function of *ERF41* in *Arabidopsis* under salt stress, potted plants were experimentally treated. *Arabidopsis* wild-type (*Col-0*) and mutant (*erf41*) plants were treated with a 300 mM salt solution. Phenotypic analysis revealed that under normal conditions, the growth status of *Col-0* and *erf41* plants showed no statistically significant difference, both of which could grow normally. After salt treatment, leaves of the *Col-0* plants exhibited wilting and drying, and the plants were nearly dead, whereas leaves of the *erf41* plants only exhibited mild wilting, with only the basal leaves withering, but they could still grow normally (Figure 6A). Then, the physiological indicators of MDA, SOD, and Pro in leaves of wild-type (*Col-0*) and mutant (*erf41*) plants under normal and salt treatment conditions were measured (Figure 6B–D). The results showed that under normal watering conditions, there were no significant differences in these physiological indicators between *Col-0* and *erf41* plants. Under salt solution treatment, the contents of MDA, SOD, and Pro in leaves of all genotypes increased, but the contents of SOD and Pro in mutant plant leaves were significantly higher than those in *Col-0* plants, while the content of MDA was significantly lower than that in *Col-0* plants. This further indicates that the ERF41 gene exerts a negative regulatory effect on salt tolerance in *Arabidopsis thaliana*.

### 3.4. ERF41 Negatively Regulates Drought Tolerance in Arabidopsis thaliana

**Figure 7:**
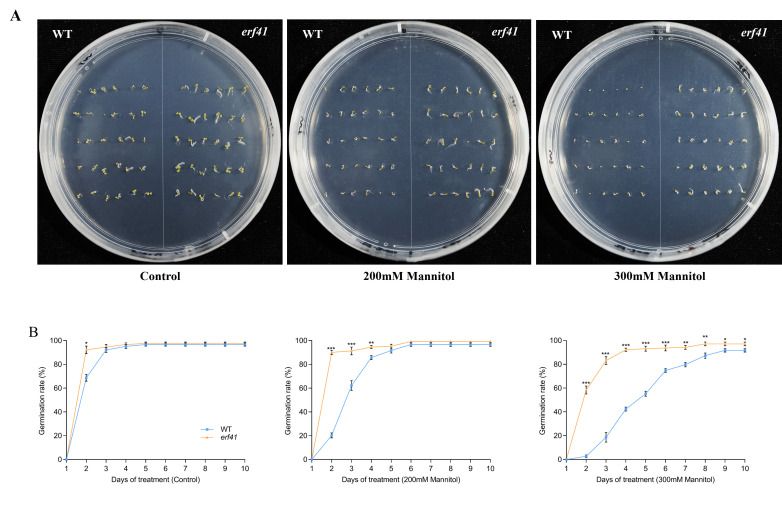
Germination conditions of WT and *erf41* mutant under drought stress. Germination performance (**A**) and germination rate (**B**) of wild-type and mutant seeds under drought stress. The data are represented as means ± SE (*n* = 30 biological replicates per genotype). The asterisks (*, ** and ***) represent significant differences (* *p* < 0.05, ** *p* < 0.01, and *** *p* < 0.001) compared with WT plants at the same time point.

To explore the function of *ERF41* in *Arabidopsis thaliana* responses to drought stress, we statistically analyzed the seed germination of wild-type (*Col-0*) and mutant (*erf41*) plants under treatments with different concentrations of mannitol (Figure 7A). The results showed that on normal 1/2 MS medium, there was no significant difference in the seed germination rate among different *Arabidopsis* genotypes, with all rates close to 100%. Under the treatments of 200 mM and 300 mM mannitol, the germination rates of *Col-0* seeds were 96.7% and 91.7%, respectively, whereas those of *erf41* seeds were 99.2% and 97.1%, which were significantly higher than those of *Col-0* seeds (Figure 7B). It was preliminarily inferred that *ERF41* exerts a function under drought stress and negatively regulates the drought tolerance of *Arabidopsis thaliana*.

**Figure 8:**
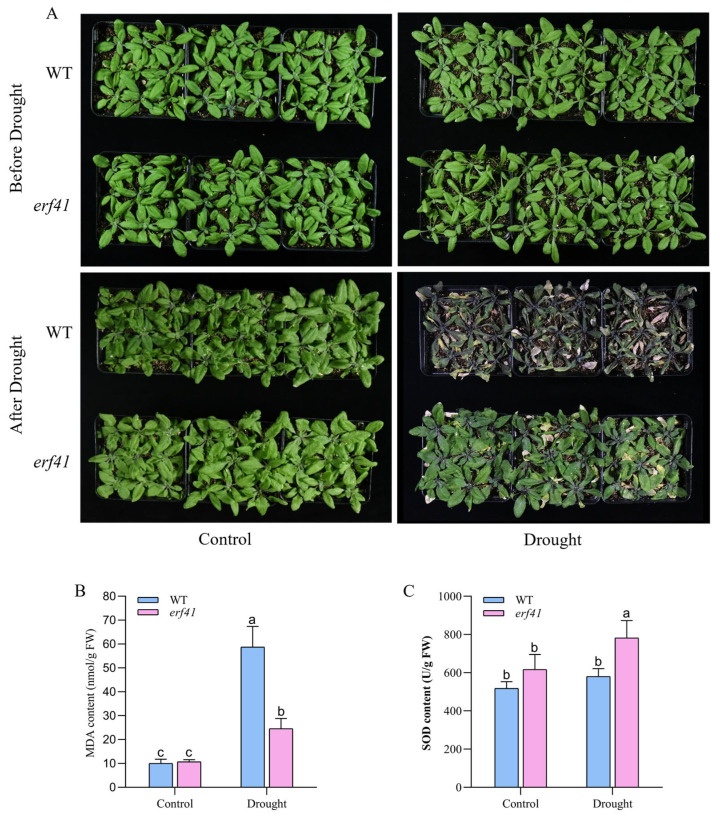
*AtERF41* negatively regulates drought tolerance in *Arabidopsis thaliana*. (**A**) Morphological differences in plants under drought experiments. The content of MDA (**B**), SOD (**C**). Data are means ± SE (*n* = 15 biological replicates per genotype). Different letters denote significant differences: *p* < 0.05.

To further explore the role of the *ERF41* gene in *Arabidopsis thaliana* responses to drought stress, wild-type (*Col-0*) and mutant (*erf41*) *Arabidopsis* plants were subjected to drought treatment. Phenotypic analysis revealed that under normal growth conditions, *Col-0* and *erf41* plants exhibited similar growth phenotypes with no significant difference, with both genotypes growing normally. After drought treatment, the leaves of wild-type *Arabidopsis* exhibited severe curling and chlorosis, followed by wilting and desiccation, and the plants were nearly dead. In contrast, the leaves of *erf41* plants showed less chlorosis than those of the wild type and maintained a much better growth status. These results indicated that the *erf41* mutant had enhanced drought tolerance under drought stress conditions. (Figure 8A). Subsequently, the physiological indices of MDA and SOD in the leaves of wild-type (*Col-0*) and mutant (*erf41*) plants were determined under both normal and drought conditions (Figure 8B,C). The results showed that there were no significant differences in these physiological indices between the leaves of *Col-0* and mutant *erf41* plants under well-watered conditions. Under drought treatment, the MDA content in the leaves of *erf41* mutants was significantly lower than that in *Col-0* plants, whereas the SOD content in the mutant leaves was significantly higher than that in *Col-0* plants. These findings demonstrate that *ERF41* plays a role in response to drought stress and negatively regulates drought tolerance in *Arabidopsis thaliana*.

## 4. Discussion

Abiotic stresses such as drought, high temperature, and high salinity are major limiting factors for global agricultural production, which inhibit plant growth and development and ultimately lead to significant reductions in crop biomass and yield [18]. To survive under adverse environmental conditions, plants have evolved sophisticated and precise regulatory networks to perceive stress signals, activate downstream stress-responsive genes, and initiate a series of physiological and biochemical responses to mitigate stress damage. The AP2/ERF transcription factors are key regulators of ethylene-responsive gene expression, participating in plant growth and development, primary and secondary metabolism, as well as responses to environmental stresses [5]. Previously, our laboratory identified an AP2/ERF transcription factor member, *Medtr3g110205*, which responds to salt-alkali stress, via transcriptome analysis of alfalfa under salt-alkali stress. In this study, we identified the homolog of *Medtr3g110205* in *Arabidopsis thaliana*, designated *ERF41*, and performed functional characterization of *AtERF41*. Conserved domain analysis of the ERF41 protein revealed that it contains one conserved AP2 domain (Figure 1E). Moreover, previous studies have demonstrated that *ERF41*, also known as *TINY2*, belongs to the dehydration-responsive element-binding protein A4 (DREB A4) subfamily within the AP2/ERF transcription factor family [13]. And, *ERF41* is induced by ABA, cold, wounding, NaCl, and drought treatments [17]. However, the function of *ERF41* in plant responses to drought and salt stresses remains unclear.

Through genetic experiments and physiological and biochemical analyses, this study found that the *Arabidopsis thaliana* mutant *erf41* exhibited enhanced tolerance to both salt and drought stresses. Under drought and salt stress conditions, plant cells rapidly produce and accumulate substantial amounts of reactive oxygen species (ROS), which induce severe oxidative damage [19,20,21]. Malondialdehyde (MDA) serves as a key physiological marker for assessing the extent of plasma membrane peroxidation [20], while proline—a widely accumulated osmolyte under such stresses—plays a critical role in maintaining protein stability [22]. Additionally, superoxide dismutase (SOD) functions to scavenge ROS generated in plants exposed to abiotic stress, thereby facilitating survival under adverse environmental conditions [23]. Therefore, these stress-related physiological indicators enable the rapid and accurate evaluation of plant tolerance to abiotic stresses such as drought and salt. In this study, compared with wild-type plants, the *erf41* mutant plants showed significantly increased SOD and proline contents and significantly decreased MDA content in leaves under both salt and drought stresses. These results provide direct evidence that *AtERF41* negatively regulates salt and drought tolerance in *Arabidopsis thaliana* (Figure 6 and Figure 8). In addition, the *erf41* mutant plants exhibited significantly improved seed germination and growth performance under salt and drought stresses, which further confirmed that *ERF41* negatively regulates salt and drought tolerance in *Arabidopsis thaliana*. Previous studies have shown that overexpression of the *TaERF6-3A* gene in *Arabidopsis thaliana* leads to the downregulation of genes related to salt stress and antioxidation, thereby negatively regulating salt and drought tolerance in *Arabidopsis thaliana* [24].

Numerous studies have demonstrated that overexpression of *OsERF19* can improve salt and drought tolerance in rice [9]. Overexpression of the *GhERF13.12* gene in *Arabidopsis thaliana* resulted in transgenic plants with stronger salt tolerance compared with the control group. In contrast, silencing of the *GhERF13.12* gene increased the sensitivity of cotton to salt stress, indicating that *GhERF13.12* positively regulates salt tolerance in both *Arabidopsis thaliana* and cotton [25]. Different from most reports that ERF transcription factors positively regulate stress resistance, the negative regulatory role of *AtERF41* reveals the functional diversity of the ERF family. *AtERF41* is closely related to TINY, which also belongs to the DREB A4 subfamily, in terms of evolutionary relationship [13]. However, *TINY* can positively regulate plant drought tolerance by activating drought-responsive genes and actively promoting ABA-mediated stomatal closure [14], whereas *AtERF41* exerts the opposite negative regulatory function. This phenomenon fully suggests that there is significant functional differentiation among members of the DREB A4 subfamily, which is worthy of further investigation. We speculate that ERF41 may directly or indirectly suppress the expression of stress-responsive genes, including those involved in ABA signal transduction, reactive oxygen species scavenging, osmotic adjustment, and stress protection. Therefore, the loss of function of *ERF41* releases this inhibition, leading to elevated expression of stress-related genes and enhanced adaptation to salt and drought stresses. Further investigation into the direct target genes of ERF41 will help clarify the precise molecular regulatory network underlying ERF41-mediated stress responses.

This study expands the understanding of the role of ERF transcription factors in plant stress responses, and proposes that ERF genes may achieve the adaptive balance of plants to environmental changes through the precise regulation of the expression of stress-responsive genes. However, this study was mainly conducted in *Arabidopsis thaliana*, and its conclusions need to be verified in more species. Future research should further explore the specific mechanism of action of *AtERF41*, including its interacting proteins and downstream target genes. Meanwhile, field trials should be carried out to evaluate the practical application effects in agricultural production.

## 5. Conclusions

In this study, we thoroughly investigated the function of the *AtERF41* gene in salt and drought tolerance of *Arabidopsis thaliana* via bioinformatics analysis, genetic experiments, and the determination of physiological and biochemical indices. Furthermore, we successfully identified and obtained homozygous *erf41* mutant plants of *Arabidopsis thaliana*. Phenotypic analysis under treatments with different concentrations of salt and mannitol revealed that the *erf41* mutant exhibited distinct growth characteristics compared with the *Col-0* under both salt and drought stresses. Specifically, the seed germination rate of the *erf41* mutant was higher than that of the *Col-0* under salt and drought stresses. Moreover, its leaves showed milder wilting and maintained normal growth after exposure to salt and drought treatments. Meanwhile, the results of physiological and biochemical index determination indicated that under salt and drought stresses, the SOD content in the leaves of the mutant plants was significantly higher than that of the wild type, whereas the MDA content was significantly lower. These findings further confirmed the negative regulatory role of the *AtERF41* gene in salt and drought tolerance of *Arabidopsis thaliana*.

## Figures and Tables

**Table 1:** Primer information.

Primer Name	Primer Sequence
BP	ATTTTGCCGATTTCGGAAC
LP	CATCTGTGGAGCTGGCTAATC
RP	GGAACGGCCGCTATACTAAAC

**Table 2:** Primer information for RT-qPCR.

Primer Name	Primer Sequence
*ERF41 *-F *ERF41 *-R *Actin *-F *Actin *-R	ATCACCACCACCACCATCAT GGGAAGTTGAGAATGGCTGC GATGCTGAGGATATTCAACCCC CCATGACACCAGTATGACGAGG

## Data Availability

The original contributions presented in this study are included in the article. Further inquiries can be directed to the corresponding author.
